# Environmentally Responsive Hydrogels and Composites Containing Hydrogels as Water-Based Lubricants

**DOI:** 10.3390/gels11070526

**Published:** 2025-07-07

**Authors:** Song Chen, Zumin Wu, Lei Wei, Xiuqin Bai, Chengqing Yuan, Zhiwei Guo, Ying Yang

**Affiliations:** 1 College of Mechanical Engineering, Hunan Institute of Science and Technology, Yueyang 414006, China; chensong@hnist.edu.cn (S.C.); weilei@hnist.edu.cn (L.W.); 2 School of Life Science, Keele University, Stoke-on-Trent ST4 7QB, UK; 3 State Key Laboratory of Maritime Technology and Safety, Wuhan University of Technology, Wuhan 430063, China; 4 Reliability Engineering Institute, National Engineering Research Center for Water Transportation Safety, Wuhan 430063, China; 5 School of Transportation and Logistics Engineering, Wuhan University of Technology, Wuhan 430063, China

**Keywords:** lubrication, water-based hydrogels, environmentally responsive, composite, friction pair

## Abstract

Both biosystems and engineering fields demand advanced friction-reducing and lubricating materials. Due to their hydrophilicity and tissue-mimicking properties, hydrogels are ideal candidates for use as lubricants in water-based environments. They are particularly well-suited for applications involving biocompatibility or interactions with intelligent devices such as soft robots. However, external environments, whether within the human body or in engineering applications, often present a wide range of dynamic conditions, including variations in shear stress, temperature, light, pH, and electric fields. Additionally, hydrogels inherently possess low mechanical strength, and their dimensional stability can be compromised by changes during hydration. This review focuses on recent advancements in using environmentally responsive hydrogels as lubricants. It explores strategies involving physical or structural modifications, as well as the incorporation of smart chemical functional groups into hydrogel polymer chains, which enable diverse responsive mechanisms. Drawing on both the existing literature and our own research, we also examine how composite friction materials where hydrogels serve as water-based lubricants offer promising solutions for demanding engineering environments, such as bearing systems in marine vessels.

## 1. Introduction

According to statistics, approximately one-third of the world’s primary energy is wasted in the process of friction. About 60–80% of mechanical components fail due to friction wear every year, and more than 50% of mechanical equipment accidents originate from lubrication failure or excessive wear. These lead to a decrease in the reliability and service life of systems and even trigger disasters. Economic losses amount to 5–7% of GDP annually in industrialized countries [1].

Lubricants are functional materials designed to reduce friction and wear between the surfaces of friction pairs, thereby enhancing mechanical efficiency and extending the service life of equipment. According to the data released by Research and Markets, the global market for industrial lubricants was estimated at USD 74.4 Billion in 2024 and is projected to reach USD 96.9 Billion by 2030, growing at a CAGR (compound annual growth rate) of 4.5% from 2024 to 2030 [2]. The core function of lubricants is to form a thin film (lubricating film) between contact surfaces, thereby reducing the friction coefficient, controlling wear, dissipating heat and preventing corrosion. From traditional mineral oils and synthetic greases to emerging water-based lubricants, ionic liquids, and nano-lubricating materials, the evolution of lubricants has closely paralleled advancements in industrial technology [3].

**Figure 1:**
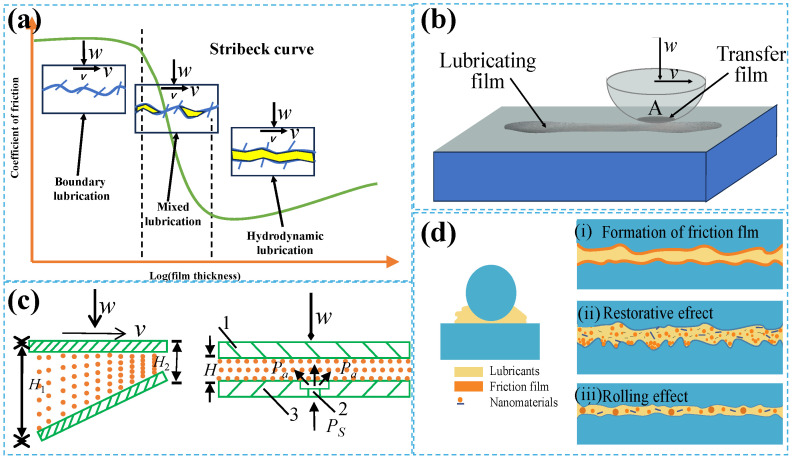
Schematics of lubrication type. (**a**) Stribeck curve; (**b**) solid lubrication; (**c**) fluid lubrication; (**d**) solid–liquid mixed lubrication. *W*: load; *v*: fluid flow rate; *H*: fluid thickness; *P*: pressure; *A*: area.

In the early 20th century, German scholar Richard Stribeck developed the well-known “Stribeck curve” by studying the friction and lubrication behavior of rolling and sliding bearings. This curve categorizes lubrication into three regimes: boundary lubrication, mixed lubrication, and fluid (hydrodynamic) lubrication, as illustrated in Figure 1a. Boundary lubrication usually occurs at low speeds, and the lubricant entering the contact area can be ignored. At this time, the asperities on the contact interface bear all the load, and the load also depends on the surface and interface film properties at the molecular scale. For hydrodynamic lubrication, the lubricating film in the contact area completely separates the two interacting interfaces, and its thickness is determined by the viscosity and entrainment speed of the lubricant. Under these conditions, the friction force depends on the rheological properties of the lubricating film in the contact area. Mixed lubrication is between boundary lubrication and hydrodynamic lubrication. The friction is determined by the boundary lubrication film and the hydrodynamic lubrication film.

Solid lubrication is realized by the application of solid lubricants between friction surfaces (Figure 1b). Solid lubrication applies two mechanisms: ① solid lubricants or their low-shear properties form a lubricating film on the friction surface, which not only has low shear strength but also adheres firmly to the substrate surface. During the friction process, the lubricating film can be transferred to the surface of the dual material in a direction to form a transfer film, so that the actual friction occurs between the transfer film and the lubricating film, significantly reducing the friction coefficient. ② This process can maintain the actual contact area of the friction pair basically unchanged. This dual action mechanism synergistically achieves efficient control of friction and wear.

Liquid and gas lubrication is classified as fluid lubrication. Fluid lubrication is mainly divided into fluid dynamic lubrication and fluid static lubrication. The principle of fluid dynamic lubrication is shown in Figure 1c. When the upper surface moves relative to the lower surface at a certain speed, the fluid between the surfaces can produce a pressurization phenomenon under the action of viscosity, thereby obtaining normal support capacity. In this process, the velocity *v* of the upper surface and the angle between the two surfaces must both exist to ensure the formation of fluid lubrication. The main working principle of fluid lubrication involves using the static pressure of the fluid to change the type of contact surface from contact to non-contact. According to the method of providing static pressure by the fluid, it can be divided into self-pressurized and externally pressurized fluid static lubrication Figure 1c. As the load-carrying capacity of liquid is much larger than that of gas, liquid lubricants are used more commonly in engineering.

By adding specific types and contents of solid materials to base oil or grease, a special solid–liquid mixed lubrication can be generated. The most common solid materials added are nanomaterials (e.g., nanoparticles, nanosheets, nanofiber, Figure 1d). A stable nano-lubricating film can be formed on the surface of the friction pair, significantly optimizing the friction performance; thus, the lubrication efficiency and component life can be improved. Its lubrication mechanism can be understood from the following perspectives: (1) nano-lubricants can form a solid lubricating film with low shear strength on the surface of the friction pair, leading a protective layer on the dual surface through the friction-induced transfer effect, so that the friction mainly occurs between the lubricating films rather than through direct contact with the substrate; (2) some nanomaterials can fill surface microcracks or defects during the friction process and form a dense repair layer under the action of friction heat or mechanical action, increasing the surface hardness and reducing wear; (3) nanoparticles can act as “micro-bearings” at the interface of the friction pair, replacing traditional sliding friction with rolling motion, thereby significantly reducing the friction coefficient.

As modern industry advances toward increasingly extreme working conditions, such as high loads, elevated temperatures, high speeds, or stringent biocompatibility requirements, the limitations of traditional lubricants are becoming more apparent. For instance, solid lubricants such as molybdenum disulfide and graphite exhibit reduced performance in humid environments, while mineral-based lubricants (e.g., oils and greases) are prone to oxidation and thermal degradation at high temperatures. Additionally, tightening environmental regulations have accelerated the demand for biodegradable and low-toxicity green lubricants.

In response, the design of next-generation lubricants focuses on several key directions: (1) composite lubrication systems, such as greases enhanced with nanoparticle additives; (2) environmentally responsive materials including smart lubricants that respond to changes in temperature, pH, or shear force; (3) biomimetic lubrication strategies, such as aqueous gels or zwitterionic polymers that mimic the properties of synovial fluid. Among these, water-based lubricants have garnered significant attention in both fundamental research and engineering applications. In recent years, water-based lubricants have become increasingly popular due to their environmental friendliness, excellent cooling properties, and biocompatibility. For example, hydrogel-based lubrication systems combining aqueous networks with functional polymers such as polyvinyl alcohol and hyaluronic acid have demonstrated unique advantages in biomedical applications (e.g., artificial joints, endoscopic coatings) and flexible electronics.

Water-based lubricants are formulated by mixing water with various additives. They are environmentally friendly, abundant in resources, and cost-effective, offering significant potential for future development. However, compared to oil-based lubricants, their lubricating performance is relatively limited, making it difficult to form a stable lubricating film. As a result, friction pairs operating with water-based lubricants often remain in mixed or boundary lubrication regimes, leading to increased friction and wear. Therefore, the lubricating performance of water-based lubricants is one of their most critical performance indicators [4]. Moreover, water-lubricated bearings often operate under complex and variable conditions, including fluctuating loads, speeds, water chemistry (e.g., pH), interface temperatures, and even alternating electromagnetic fields. For instance, in ship propeller bearings, the load and speed typically range from 1 to 20 MPa and 10 to 2000 rpm, respectively. Water quality also varies significantly depending on the operating environment. In freshwater, the pH ranges from 6.0 to 8.5 and often contains high levels of particulate pollutants such as silt, with particle sizes ranging from 1 to 100 μm and concentrations reaching or exceeding 5%. In contrast, seawater has a pH range of 7.5 to 8.4 and generally contains fewer particulates, especially in open-sea conditions. Additionally, frictional heat can raise the local contact temperature of water-lubricated bearings to as high as 80 °C [5].

Given these challenges, the stability and environmental responsiveness of water-based lubricants under varying operating conditions are also key performance metrics. Recent advancements have enabled the development of self-responsive water-based lubricants through the optimized formulation of additive types and concentrations. These lubricants can adapt to changes in the working environment, for example, by adjusting viscosity in response to shear force or temperature, thereby maintaining effective lubrication and expanding their potential applications [6]. In certain tribological applications, water lubrication is often necessary or highly desirable. However, due to water’s low viscosity and high fluidity, it struggles to form a stable lubricating film. Under extreme stress conditions, relying solely on a water layer as a lubricant is often impractical. Studies have shown that combining water with surface-grafted polymers, both biological and synthetic and featuring diverse structural and chemical properties, can significantly reduce interfacial friction and even achieve super-lubricity. These findings provide strong theoretical support for the design and development of advanced lubricating materials. Among these, hydrogels have emerged as one of the most promising candidates for water-based lubrication.

Hydrogels are composed of a three-dimensional polymer network with water as the solvent. They are a unique class of soft materials formed by cross-linking hydrophilic polymers through physical, chemical, or combined methods. This network structure allows hydrogels to absorb and retain large amounts of water without dissolving. Therefore, hydrogels can be used in underwater equipment. However, few thorough reviews have been conducted in this specific field previously.

Within the polymer matrix, water exists in various forms, including bonded water, bound water, and free water, each of which contributes differently to the material’s lubrication and mechanical properties. Hydrogels can be composed of either single or multiple cross-linked polymers. Based on the origin of the polymers used, hydrogels are generally classified as either natural or synthetic, and they can be formed from homopolymers or copolymers.

Natural polymers used in hydrogel preparation are primarily polysaccharides and proteins. Polysaccharides contain numerous functional groups along their molecular chains that can readily participate in the formation of dynamic covalent or non-covalent bonds, making them well-suited for hydrogel formation. Common polysaccharide-based hydrogel materials include cellulose, chitosan, alginate, and hyaluronic acid. Proteins, which are biomacromolecules composed of amino acids linked by peptide bonds, also serve as excellent hydrogel precursors. Their molecular chains contain polar groups (e.g., –OH, –NH_2_, –COOH), non-polar side chains (e.g., leucine, phenylalanine), and regions with opposite charges that can engage in non-covalent interactions. These features, along with chemical reactions between functional groups, facilitate protein cross-linking. Common protein-based hydrogel materials include silk protein, collagen, serum albumin, whey protein, and soy protein. Naturally derived hydrogels offer significant advantages for biomimetic applications, such as soft robotics for patient care, due to their excellent biocompatibility and biodegradability. However, they often suffer from poor load-bearing capacity, batch-to-batch variability stemming from their biological origin, and limited tunability in terms of structure and properties.

In contrast, synthetic polymer-based hydrogels offer advantages such as enhanced chemical stability, superior mechanical properties, and ease of preparation. By modifying the polymer backbone or monomer units, synthetic hydrogels can be engineered to exhibit a broader range of physicochemical properties than their natural counterparts. The synthetic polymers commonly used in hydrogel preparation include polyvinyl alcohol (PVA), polyacrylamide (PAM), polyacrylic acid (PAA), polyethylene glycol (PEG), and polyvinyl pyrrolidone (PVP). Generally, hydrogels made from homopolymers tend to exhibit relatively poor mechanical strength. However, their mechanical properties can be significantly enhanced by incorporating nanomaterials such as nanofibers and multi-walled carbon nanotubes or by constructing multi-network structures through the cross-linking of multi-component polymers. In general, synthetic polymer hydrogels exhibit high mechanical strength and elasticity, making them well-suited for load-bearing applications. Their structures can be precisely engineered, allowing for the customization of properties, including stimuli responsiveness, to meet specific functional requirements.

To address existing knowledge gaps and recent advancements in the use of hydrogels as water-based lubricants, this review aims to explore specific hydrogel systems that operate effectively in dynamic environments, an area that has not been thoroughly examined in previous literature. In particular, the objective of this study is to provide a systematic overview of the classification, working mechanisms, and recent research progress of environmentally responsive hydrogels used as water-based lubricants. For coherence and logical flow, the review is structured into two main sections. Section 2 focuses on hydrogels that respond to various environmental stimuli such as mechanical stress, temperature, pH, light, and chemical signals. These responsive systems offer valuable design strategies for applications in intelligent soft robotics, biomedical catheters, underwater mechanical transmissions, and actuators. Section 3 investigates the use of hydrogel-based composites as water-based lubricants in metallic tribological systems. It highlights strategies to overcome the inherently low mechanical strength of conventional hydrogels, which is a critical limitation in high-load metallic applications, for instance, water-lubricated bearing in ships. The review concludes with a summary of structural design strategies for environmentally responsive hydrogels and offers perspectives on future research opportunities in the field of water-based lubrication.

## 2. Environmentally Responsive Hydrogels

As hydrogel research continues to progress, the focus has shifted from traditional formulations to environmentally responsive hydrogels, a class of polymer materials capable of intelligently responding to subtle changes in the external environment. These materials, also known as environmentally responsive hydrogels or supramolecular gels, can undergo specific physicochemical changes when exposed to targeted external stimuli. Compared to conventional hydrogels, environmentally responsive hydrogels exhibit reversible and controllable responses to external triggers. These responses can lead to significant changes in the hydrogel’s properties, such as the assembly or disassembly of the polymer network, often resulting in a phase transition between gel and sol states.

Responses to the environment can be broadly categorized into two types: (1) responses to chemical factors, including pH changes, ionic strength, and specific chemical agents; (2) responses to physical factors, such as temperature, light, electric fields, and mechanical forces. As a relatively new class of materials, environmentally responsive hydrogels hold great promise for practical applications and future development. In recent years, they have begun to attract attention in the field of tribology, particularly within mechanical engineering and bioengineering, where their adaptive and responsive properties offer exciting new possibilities.

On the other hand, hydrogels inherently possess relatively poor mechanical strength, making them unsuitable for withstanding high loads. Additionally, they undergo significant dimensional changes upon swelling. When used as additives, this swelling can adversely affect the dimensional stability of composite polymer materials. As a result, hydrogel materials still face several challenges in engineering applications. Water-lubricated bearings, for example, operate under complex and variable conditions, including fluctuating loads and speeds, changing water chemistry, and varying interface temperatures. To meet the demanding requirements of such environments, intelligent responsive hydrogels must exhibit properties such as load-bearing capacity, speed adaptability, pH stability, and temperature responsiveness.

Several strategies have been explored to address these challenges. Shear-responsive hydrogels are designed to form a lubricating layer under high shear rates, enabling friction-induced responsiveness [7,8,9]; hydrogels that are responsive to pH and ionic strength are tailored for seawater environments. These hydrogels respond dynamically to changes in the charge state at the lubrication interface [10,11,12]; temperature-responsive hydrogels are engineered to undergo localized thermosensitive phase transitions, adapting to temperature fluctuations during operation [13,14,15]. These advancements highlight the potential of environmentally responsive hydrogels to adapt to the multifaceted demands of water-lubricated systems, paving the way for broader engineering applications.

### 2.1. Shear-Responsive Lubricating Hydrogels

Shear force arises when there is a velocity gradient between adjacent layers of fluid. Within a friction pair, shear force represents the dissipation of interfacial interaction energy. During friction, relative motion occurs between the surfaces of the friction pair, the molecular layers of the lubricant, resulting in a shear effect. That is because the lubrication state is determined by quantitatively calculating the minimum thickness of the molecular layers of the lubricant, and the calculation formula is as follows [16]:

(1) $$h_{min}=3.63{\left(\alpha E^{\prime }\right)}^{0.49}{\left(\frac{\eta U}{E^{\prime }\gamma }\right)}^{0.68}{\left(\frac{P}{E^{\prime }\gamma }\right)}^{-0.073}\left(1-e^{-0.68k}\right)$$

where *r* is the radius of the ball model of the asperity on dual surface, *α* is the viscosity–pressure coefficient, *E*′ is the effective elastic modulus, *η* represents the dynamic viscosity of the hydrogel, *U* is the average sliding velocity, *P* is the load, and *k* is the elliptical parameter.

In the absence of lubrication or under boundary lubrication conditions, surface asperities on the friction pair interlock mechanically due to surface roughness and intermolecular adhesion. These interactions are governed by van der Waals forces or chemical bonds. In boundary lubrication, lubricant molecules are adsorbed onto the surface, and the resulting resistance depends on the strength and arrangement of the molecular chains. In contrast, under hydrodynamic or elastohydrodynamic lubrication, the friction surfaces are fully separated, and shear force arises from the viscous flow of the lubricant. The magnitude of this shear force is influenced by factors such as material strength, surface roughness, lubrication conditions, and operating parameters. If the normal pressure in the vertical direction and shear force in the horizontal direction are extracted, the friction coefficient of the shear progress can be obtained using the following equation [17]:

(2) $$\mu =F_{f}/F_{n}$$

where *μ* is the friction coefficient, *F_f_* is the normal pressure in the vertical direction, and *F_n_* is shear force in the horizontal direction

Shear-responsive hydrogels can provide good protection through long-term lubrication, providing a new way to lubricate rubbing parts. However, due to the difficulties of controlling the shear force, the development of shear-responsive hydrogels is limited. At present, there are only a few research reports of shear-responsive hydrogels [18].

**Figure 2:**
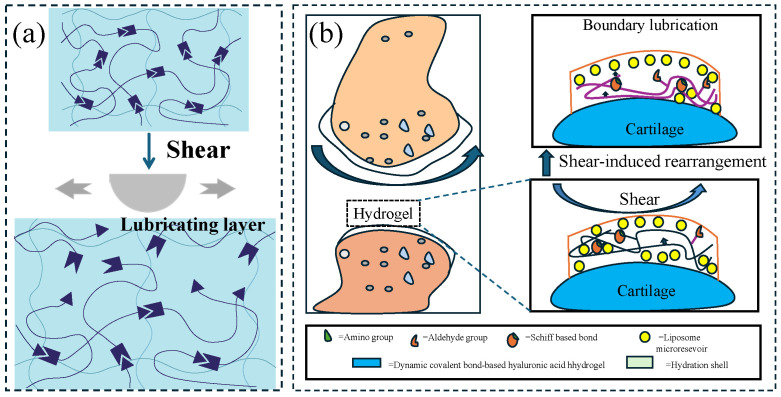
Schematics of shear-responsive lubricating hydrogels. (**a**) Shear-responsive lubrication mechanism: Under shear force, the interaction between the segments is destroyed and a lubricating layer is formed. (**b**) CLX@Lipo@HA gel can expose the internal liposome micro reservoirs on the outer surface through shear-induced structural rearrangement to form a boundary layer, figure redrawn from Lei et al. [27], under CC BY license.

The primary lubrication mechanism involves shear thinning or the dissociation and reassembly of non-covalent supramolecular chains under shear stress, leading to the formation of a lubricating layer (Figure 2a). When shear force is applied, the hydrogel network undergoes reversible cross-linking or molecular chain alignment, facilitating the release or directional arrangement of interfacial lubricants. This results in a significant reduction in the friction coefficient from static values (0.3–1.0) to dynamic values (0.01–0.1). By tailoring the types of dynamic bonds (e.g., reversible covalent bonds for enhanced durability) and optimizing network topology (e.g., double-network structures for improved mechanical strength), these hydrogels can achieve excellent fatigue resistance (over 10^4^ cycles) and environmental stability (an elastic modulus ranging from 10 kPa to 1 MPa). These properties make them particularly well-suited for applications requiring adaptive friction control, e.g., underwater robotic joints, artificial cartilage, and marine equipment protection.

Articular cartilage is a special porous substance with the thickness of 1–4 mm, and the cavities within it are filled with liquid. When subjected to load, this liquid is affected by pressure and begins to move towards the joint surface and penetrate to the outer surface of the joint to alleviate this pressure effect, thereby acting as a lubricant. This lubrication mechanism is called exudative lubrication, maintaining the friction coefficient of the articular cartilage between 0.012 and 0.015. At the same time, the flow of this liquid within and outside the cartilage forms a film composed of fluid between the joints, which is also beneficial for the joints. When the joint is not bearing load, the liquid is stored in the porous cartilage; meanwhile, when pressure load acts on the cartilage, the stored liquid continuously flows out from the cavities, partially counteracting the exudative effect, and, by increasing the thickness of the membrane between the joints, it plays a role in improving lubrication.

Inspired by the lubrication phenomenon of articular cartilage, Zhang [19] combined a thixotropic supramolecular FT (friction modifier) and PAAm (polyacrylamide)/PVA (poly(vinyl alcohol)) dual network to prepare a shear-force-responsive supramolecular lubricating hydrogel. During the shear process, the FT supramolecular hydrogel partially disassembled and seeped out to the hydrogel surface, causing the friction coefficient to drop from 0.0372 ± 0.0007 to 0.0233 ± 0.0021. Fluorescence spectroscopy detected the disassembled FT on the hydrogel surface, and its fluorescence intensity decreased with the increase in shear cycles, indicating that FT gradually seeped out during the lubrication process and participated in lubrication. This research brings a new perspective to the design of artificial lubricated joints.

However, achieving self-healing in a complex dynamic shear environment remains a challenge. Zhang [20] used PHEAA (poly(2-hydroxyethyl acrylamide)) with self-healing abilities to replace PAAm, combined with FT and PHEAA/PVA to prepare a self-healing semi-convertible hydrogel (SHSCH). The shear-responsive lubrication function originated from the disassembly of FT, while the self-healing function originated from the large number of hydrogen bonds within PHEAA and the reassembly of the sol-state FT monomers flowing in the scratch. During the shearing process, the friction coefficient of SHSCH decreased from 0.22 ± 0.01 to 0.09 ± 0.01. Compared with the static recovery efficiency of SHSCH, the self-healing efficiency under dynamic shear conditions was 64.97%, which was much higher than the 38.57% of static self-healing (the repair time was 240 min). The shear angle also had a significant effect on self-healing. When the shear angle was 0° and 30°, the self-healing stress of SHSCH was 106.49 ± 12.97 kPa and 84.42 ± 14.87 kPa, respectively, which was higher than the value of 63.22 ± 5.12 kPa for static self-healing. This study provides a promising basis for designing self-healing soft materials under dynamic stimulation.

Inspired by the lipid layer of articular cartilage, Lin et al. [21] prepared a PHEMA (poly(hydroxyethylmethacrylate)) lubricated hydrogel containing PC (phosphatidylcholine) lipids. This trace lipid concentration can form a lipid boundary layer of molecular thickness and can continuously self-renew. Compared with hydrogels without PC lipids (*μ* was 0.5~1), the friction coefficient of the hydrogel containing lipids was lower, about 0.02 at low loads and about 0.005 at high loads. Its lubrication mechanism can be attributed to the synergistic effect between the hydration of the PC lipid head group and the stability of the PC lipid bilayer. At the same time, this lipid-based boundary layer can be rebuilt after wear by gradually releasing lipids, and this lubrication effect still exists when the hydrogel is dried and rehydrated. This hydrogel provides a new technology for preparing self-lubricating, low-friction, and low-wear hydrogels. It has been reported that liposomes play a key role in lubrication. In order to overcome the problem of damage to the surface liposome coating after friction, Lei [22] prepared hyaluronic-acid-based hydrogel microspheres (RAPA@Lipo@HMs) and explored their lubricating effect. The microspheres had a self-renewable hydration layer. This self-renewable hydration layer is formed by friction and the wear of the exposed liposomes, thereby improving lubricity. The friction coefficient of the newly prepared Lipo@HMs was about 0.04 because there were few liposomes on the outermost surface. As the friction test progressed, more liposomes were exposed to the surface under friction, forming a hydration layer, and the friction coefficient decreased and stabilized at around 0.03. Subsequently, as Lipo@HMs were squeezed out and water was lost, the friction coefficient began to increase. RAPA@Lipo@HMs can provide effective lubrication and may alleviate friction-related diseases such as osteoarthritis.

Inspired by the soft–hard combination strategy of biomatrix, Fu [23] mixed graphene oxide (GO) into a supramolecular hydrogel (cyclodextrin-based PPR (polyrotaxane-based polymer)) and infiltrated it into textured HA (hydroxyapatite) composites to prepare a shear-responsive lubricating hydrogel coating. Due to the friction behavior, the hybrid hydrogel stored in the textured HA underwent a gel–sol transition, and the sol-state hydrogel moved toward the surface, forming a thin film on the worn surface. In addition, the cyclodextrin-based PPR supramolecular structure can adsorb water molecules to form a hydration layer, thereby improving lubricity and reducing wear. Compared with the textured HA coating, the prepared hydrogel coating reduced the friction coefficient from 0.43 to 0.089. This hybrid hydrogel coating broadens the application of PPR supramolecular hydrogels in the field of biomedical materials.

Torres [24] prepared emulsion microgel particles for use as bio-lubricants. The mechanism of their low friction coefficient may be the synergistic release of the hydrogel particles triggered by enzymes and shear, thereby improving lubrication. Wang [25] prepared a semisolid supramolecular hydrogel of 2,4,6-triamino-1,3,5-triazine-1-ium diisopentyl phosphate. During the friction process, the destroyed hydrogel can be adsorbed on the friction interface to form a lubricating film, thereby effectively reducing the interfacial friction. Ha [26] prepared an AgNP (silver nanoparticle)-modified PEG/α-CD (poly(ethylene glycol)/α-cyclodextrin) supramolecular hydrogel coating to simulate the exudative lubrication mechanism of cartilage. This supramolecular hydrogel exhibits thixotropic and temperature-responsive gel–sol transition behavior, so it can exude to form a sol layer under specific stimulation and functionalize it into a responsive synovial fluid to achieve lubrication.

Lei [27] combined CLX (celecoxib)-loaded liposomes with hyaluronic-acid-based hydrogels (CLX@Lipo@HA), and the prepared hydrogels had the characteristics of shear-responsive lubrication. Under the action of shear force, the liposomes stored in the HA matrix would aggregate on the surface of the hydrogel to form a boundary lubrication layer, providing stable lubrication (Figure 2b). The average friction coefficient of the substrate in PBS (phosphate-buffered saline) solution (0.062 ± 0.007) was much higher than that of HA gel (0.041 ± 0.004) and Lipo@HA-gel (0.031 ± 0.003); the produced wear width on Lipo@HA-gel (9.87 ± 1.80 μm) was much lower than that of the HA gel (17.64 ± 3.15 μm) and the substrate in the PBS solution (27.51 ± 3.62 μm). In addition, the friction coefficient–time curve of the HA gel first decreased and then increased. The mechanism could be the lubricating film being destroyed as the friction test proceeded; meanwhile, the friction coefficient–time curve of Lipo@HA-gel had only a short increase and then a continuous decrease. This may be due to the exposure of liposomes on the gel surface, forming a boundary lubrication layer. Inspired by the composition and function of the meniscus, Liu et al. [28] prepared a self-lubricating and friction-responsive gelatin hydrogel. This hydrogel was enriched with nanoliposomes and loaded with DS (dermatan sulfate) for anti-inflammation and KGN (Kartogenin) for cartilage regeneration. The enriched liposomes can form a hydration layer to reduce friction. In addition, the hydrogel can release liposomes upon undergoing friction, which can be observed using laser confocal scanning microscopy. Furthermore, DS and KGN were also released, thereby reducing inflammation and improving cartilage regeneration.

Unlike the above reports that the shear-induced disassembly of chain segments forms a lubricating layer, thereby reducing friction, Cao [29] proposed a PAA-co-PAM (poly (acrylic acid-co-acrylamide)) hydrogel that was gelled by shear. Under the action of shear, the polymer chain segments became ordered, thereby increasing the strength of the hydrogen bonds between the chains and forming a gel. The lubricating film formed on the Si3N4 surface transformed the hard–hard contact into a soft–soft contact, thereby reducing friction and wear.

### 2.2. Temperature-Responsive Lubricating Hydrogel

**Figure 3:**
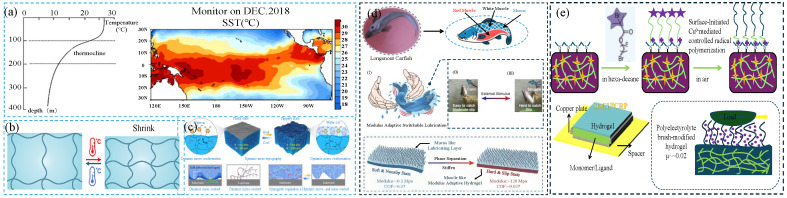
Schematics of temperature-responsive lubricating hydrogel. (**a**) The variation of the water temperature against the water depth in ocean (Seawater temperature varies with region and depth), data from [30]. (**b**) Temperature-responsive lubrication mechanism: e.g., PNIPAAm hydrogel shrinks above LCST. (**c**) Stick-slip switching mechanism of DMCS hydrogel and the relationship between temperature and adhesion strength or friction of DMCS hydrogel, figure adapted from Zhang et al. [32] under CC BY license. (**d**) Design concept of MALH, figure adapted from Zhang et al. [33] under CC BY license. (**e**) Preparation of polymer-brush grafted hydrogel surface and COF of hydrogel, figure adapted from Zhang et al. [41] under CC-BY-NC-ND license.

The water temperature gradually decreases with the increase in the depth of the ocean. For every 1000 m of depth, the temperature drops by 1–2 °C [30] (Figure 3a). When the depth reaches 1000 m, the water temperature is about 4–5 °C; at a depth of 2000 m, it is 2–3 °C; after exceeding 3000 m, the water temperature further drops to 1–2 °C. In the range of 3000 to 4000 m, the sea water temperature can reach 2–−1 °C.

Temperature-responsive lubricating hydrogels are a smart lubricating material whose tribological properties can be dynamically regulated with changes in ambient temperature; they exhibit excellent adaptive abilities in water environments. This type of gel is usually composed of thermosensitive polymers. Typical thermos-responsive polymers include poly(N-isopropylacrylamide) (PNIPAm), poly(dimethylaminoethyl acrylate) (PDMAEMA), poly(ethylene glycol) methyl ether methacrylate) (PDEGMA), and poly(N-vinylcaprolactam) (PNVCL). They can trigger the hydrophilic–hydrophobic phase transition of the polymer chain through temperature changes, thereby reversibly adjusting the surface hydration state and lubrication properties. When the temperature is below the critical solution temperature (LCST), the gel remains highly hydrated and exhibits low-friction characteristics; when the temperature exceeds the LCST, the polymer network undergoes hydrophobic contraction, resulting in a thinning of the surface hydration layer and a corresponding increase in the friction coefficient. Through molecular design (such as introducing zwitterionic monomers or constructing interpenetrating networks), hydrogels’ temperature-sensitive response range (adjustable from 20–40 °C) and mechanical strength (elastic modulus can reach MPa level) can be optimized, making them suitable for fields requiring temperature-adaptive lubrication, such as biomedical catheters and underwater mechanical transmissions.

As shown in Figure 3b, temperature-responsive polymers generally have a lower critical solution temperature (LCST) or an upper critical solution temperature (UCST). When the ambient temperature is lower than the lower critical solution temperature, the main interaction force between the hydrophilic segment and the water molecules is hydrogen bonding. The hydrogel is highly swollen and hydrophilic. The stable hydration layer on the surface gives it excellent lubricity but weak load-bearing capacity. In the critical phase transition range (T ≈ LCST, 32–35 °C), the hydrogel undergoes a dynamic phase transition, resulting in the destruction of the hydration layer, a sharp drop in lubrication performance and the occurrence of the stick-slip phenomenon. In the high temperature range (T > UCST, > 35 °C), the hydrophobic interaction between hydrophobic segments is enhanced, the hydrogen bonds are weakened, the hydrogel shrinks violently and becomes hydrophobic, the lubrication performance deteriorates significantly but the mechanical strength improves; extreme high temperatures (T ≫ UCST, > 50 °C) lead to structural destruction and the complete failure of lubrication.

Ma [31] prepared a hydrogel actuator with an asymmetric structure (PNIPAAm/PAAc-Fe) and studied its lubrication properties and potential applications. One side of this hydrogel was an unstructured PNIPAAm/PAAc-Fe hydrogel, and the other side was an ordered PNIPAAm/PAAc-Fe hydrogel column. The hydrogel actuator can switch the friction and adhesion of the surface and generate actuation force when the temperature changes or the solvent is exchanged (ethanol and water). Its response mechanism may be an asymmetric stress difference. As the temperature changes, the friction of the hydrogel also changes accordingly. Below the LCST of PNIPAAm, the prepared hydrogel was in a swollen state and therefore showed lower friction. Above the LCST, the hydrogel exhibited high friction due to the collapse of the PNIPAAm chains. The friction can be switched between 40 °C and 9 °C, demonstrating its temperature-responsive lubrication characteristics. Combining thermal-responsive behavior and switchable friction, the hydrogel actuator can easily grasp and release plastic balls, as well as grasping and lifting screws, other materials, and oil droplets, providing a new design strategy for intelligent soft robots.

Zhang [32] prepared a switchable adhesion DMCS (dynamic multiscale contact synergy)-hydrogel ((p(AAm-co-AAc-co-NIPAAm-co-DMA), Acrylamide (AAm), acrylic acid (AAc), N-isopropyl acrylamide (NIPAAm), dopamine methac-rylamide (DMA)) through dynamic multiscale synergy and studied its regulation mechanism. The hydrogel can quickly switch between the slippery state and the sticky state (Figure 3c). Below the LCST of the DMCS-hydrogel, the N-isopropyl group can form intermolecular hydrogen bonds with the adjacent water molecules, locking the water in the hydrogel, so that the surface of the DMCS-hydrogel is filled with air. The non-polar part of the hydrogel skeleton and the catechol group is exposed to the air, resulting in molecular-scale adhesion on the hydrogel surface. From a macroscopic point of view, the hydrogel has high adhesion and low friction. Above the LCST, the intramolecular hydrogen bonds of the hydrogel were destroyed, and a large number of water molecules overflowed to the surface of the hydrogel, resulting in the rearrangement of hydrophobic and hydrophilic groups and the migration of carboxyl groups. Therefore, the catechol groups cannot adhere to the substrate, and the hydrogel has low adhesion and high friction. This hydrogel had the characteristics of dynamically regulating adhesion and friction and can be widely used in programmable adhesive materials and smart devices. Zhang [33] simulated the muscle-hardening mechanism of catfish and prepared a modulus-adaptive hydrogel with lubrication ability (poly (AAcCaAc-co-HEMA-Br-PSPMA) hydrogel, MALH (modulus-adaptive lubricating hydrogel)). It consists of a mucus-like hydrophilic lubricating layer on the top and a muscle-like hydrogel on the bottom. Through thermally triggered phase separation, the hydrogel achieves a transition from a soft/high friction state (load *p* = 0.3 MPa, friction coefficient *μ* = 0.37) to a hard/lubricated state (*p* = 120 MPa, *μ* = 0.027) (Figure 3d). This modulus adaptation mechanism originates from the electrostatic interaction of the hydrophobic medium at high temperatures. Under high temperature conditions, the hydrophobic residue (acetate) dehydrated and formed a hydrophobic region, thereby reducing the dielectric constant, causing phase separation and strengthening the electrostatic effect, resulting in the rapid switching of MALH from a soft gel state to a hard plastic state. This hydrogel with switchable adhesion and friction has potential applications in the fields of smart sports equipment and soft robots.

The poly(NIPAAm-AAm)-GO composite hydrogel prepared by Wu [34] exhibited temperature-controlled tribological behavior. Above the LCST, the composite hydrogel was in a contracted state and exhibited high friction (> 0.2), but, below the LCST, the composite hydrogel in a swollen state had lower friction (< 0.03), and this temperature-controlled friction behavior was reversible. Based on this adjustable tribological property, the composite hydrogel tweezers can easily clamp a hot steel ball, but it has difficulty in clamping a cold steel ball. The iron block can be attracted by the magnetic field and move when below the LCST, but it cannot be attracted by the magnetic field and move when above the LCST. This phenomenon occurred because the friction was small at low temperatures and large at high temperatures. Liu [35] prepared PNIPAAm-g-PEG temperature-responsive microgels. Compared with pure water, the introduction of microgels can effectively reduce the friction coefficient. In addition, when the temperature raised from 25 °C to 50 °C (the load is 25 N), the microgels adsorbed on the surface increase, thereby improving the boundary lubrication effect and reducing the friction coefficient. When BTA (benzotriazole) was introduced into the microgel, the friction coefficient decreased with increasing temperatures when the load was 25, 50, and 100 N. This may be because the hydrophobic interaction between BTA and microgels enhanced the lubrication effect, so the temperature-responsive lubrication is more obvious. When PSPMK (poly (3-sulfopropyl methacrylate potassium salt)) brushes were grafted onto PNIPAAm microgels [36], the prepared microgels had ultra-low friction coefficients and drug-release performance due to the hydration lubrication effect of PSPMK brushes and the temperature responsiveness of PNIPAAm microgels. However, for PNIPAAm-Br microgels, the hydrophobic transition of PNIPAAm with the increase in temperature led to an increase in the friction coefficient.

Inspired by the movement of worms, Vernerey [37] proposed a temperature-responsive PNIPAAm hydrogel particle that can move in a closed channel. Its movement mechanism was a combination of periodic deformation (heating or cooling) and asymmetric friction with the surrounding environment. Xu [38] prepared a thermosensitive PNIPAm microgel used as lubricant additives, and the thermal tribological behavior of the 316L stainless steel/ultra-high-molecular-weight polyethylene contact surface under water lubrication conditions was investigated. Tribological tests showed that, as the test temperature increased to about 32 °C, the microgel particles dehydrated and collapsed, and the spherical structure of the microgel changed, resulting in an irregular shape, at which time the friction coefficient was high. Above the LCST, the microgels shrank, became dense, and formed a spherical shape again, promoting the rolling and sliding of the particles, resulting in a decrease in the friction coefficient. The skin of humans and some animals changes its surface morphology when stimulated by the external environment. Inspired by goose bumps, Li [39] prepared a thermally responsive poly (NIPAm-co-AA) microgel particle array. The change in particle morphology between 25 °C and 37 °C will lead to the change of adhesion and friction. At 25 °C, poly(NIPAm-co-AA) microgel was in a swollen state. The microgel in the swollen state is easy to deform and has a large effective contact area, resulting in high adhesion; however, the high hydration of the polymer chain and the presence of a water film led to low friction. When the temperature raised to 37 °C, the surface morphology of the microgel shrank and the contact area decreased, resulting in low adhesion; the destruction of hydrogen bonds and dehydration led to high friction.

Liu [40] prepared a double-layered thermo-shrinkable [P(NIPAAm-AA-iBr/Fe^3+^)] hydrogel and grafted PSPMA polyelectrolyte brushes onto it. The top hydrogel/brush composite layer had lubricating properties, while the bottom thermo-shrinkable hydrogel layer had load-bearing capacity and exhibited a modulus that was tunable with temperature. Above the LCST, the friction of the top composite layer decreased due to the increase in the modulus of the bottom hydrogel. When Fe_3_O_4_ nanoparticles were introduced into the material, the in situ improvement in lubrication can be achieved under near-infrared (NIR) light irradiation. This lubrication-regulated behavior can be attributed to the synergistic effect of the increased load-bearing capacity of the bottom layer and the enhanced lubrication behavior of the top layer with the increase in the density of the polyelectrolyte brush chains, similarly to the adaptive lubrication mechanism of the natural cartilage layer. This research provides inspiration for new biomimetic lubricating materials and a strategy for designing smart and stable friction-driven devices. Zhang [41] used surface-initiated controlled radical polymerization mediated by Cu^0^ (Si-Cu^0^ CRP (controlled radical polymerization)) to graft polymer brushes onto the hydrogel surface. Compared with bare hydrogel, the hydrogel grafted with polymer brushes had a lower friction coefficient (Figure 3e). Different polymer brushes gave hydrogels different properties. For PNIPAAm brushes, the prepared composite hydrogel had temperature-responsive properties. When the temperature was 40 °C, the conformation of the PNIPAAm brush collapsed, causing the friction coefficient to increase from 0.05 at 20 °C to 0.23 at 40 °C, and this transition was reversible. Chen [42] introduced Tween 80 (T80) into PVA hydrogel to prepare a super-lubricating hydrogel. At a low sliding speed (0.01 mm/s), the hydrogel had an ultra-low friction coefficient of 10^−3^ to 10^−4^. This ultra-low friction coefficient came from the rough surface formed by Tween 80 and the hydrophobic mold, as well as the high carbon content on the hydrogel surface. In addition, the friction coefficient (COF) of the hydrogel was also affected by temperature. The high temperature produced stronger boundary lubrication, resulting in a decrease in the friction coefficient. The friction coefficients of PVA (poly (vinyl alcohol))-H_2_O/GC/T80-Glass and PVA-H_2_O/GC/T80-PTFE (polytetrafluoroethylene) decreased from 2.5 × 10^−3^ to 1.0 × 10^−3^ and from 2.0 × 10^−3^ to 5.0 × 10^−4^, respectively. Interestingly, the hydrogel fragments could be reconverted into a mixture solution after heating at 95 °C for 30 min, and the solution could be reshaped into a hydrogel.

### 2.3. Photothermal Responsive Lubricating Hydrogel

The transformation of light and heat signals in seawater involves two main processes: natural processes and human influences. In natural processes, solar radiation (especially short-wave blue-green light) is partially converted into thermal energy when penetrating seawater, causing the surface water temperature to rise, driving thermal convection and stratification. While the photosynthesis of plankton converts light energy into chemical energy, bioluminescence (such as the blue light of dinoflagellates) reflects the conversion of chemical energy to light energy in organisms. Waste heat introduced by human activities such as ship emissions and submarine cables, as well as artificial light sources (such as LEDs) locally change the thermal structure of the water and interfere with the light-response behavior of organisms.

**Figure 4:**
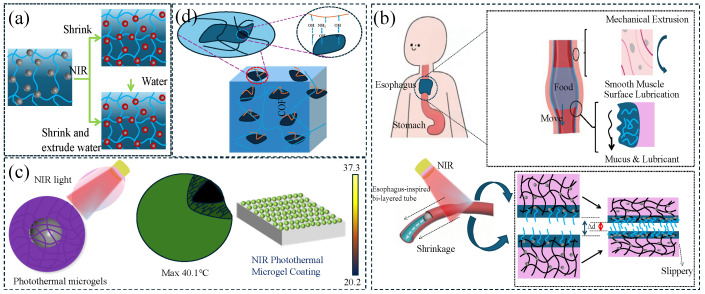
Schematics of photothermal responsive lubricating hydrogel. (**a**) Photothermal responsive lubrication mechanism: Contraction of PNIPAAm photothermal sensitive hydrogel. (**b**) Esophagus-inspired tubular soft actuator, figure adapted from Liu et al. [45] under CC BY license. (**c**) Schematic diagram of PTMGs for interfacial friction control & infrared thermal image of microgel coating and the change of COFs of microgel coating with load at different temperatures, figure adapted from [48,49] with the journal’s permission. (**d**) Composition of hydrogel and the change of volume and COF in photothermal response, figure adapted from Wu et al. from [50] under CC BY license.

Photothermal responsive lubricating hydrogel is a smart material that regulates lubrication properties through light and is particularly suitable for on-demand friction control in water environments. Photothermal response is a special temperature response method that requires photothermal particles to convert light energy into heat energy. This type of gel is usually constructed by combining photothermal conversion components (such as polydopamine, gold nanorods or graphene) with thermosensitive polymers (such as PNIPAM), and it uses the photothermal effect to achieve non-contact regulation. Under near-infrared light irradiation, photothermal materials convert light energy into heat energy, causing a local temperature rise, which in turn triggers the hydrophilic and hydrophobic phase transition of the thermosensitive network, dynamically regulating the thickness of the surface hydration layer and lubrication properties (the friction coefficient can be reversibly changed in the range of 0.01–0.5). However, the main body of photothermal responsive lubricating hydrogels is still mostly based on polymer materials with a temperature phase transition, such as PNIPAAm hydrogel, so its lubrication mechanism is similar to the temperature-responsive lubrication mechanism: that is, the hydrogel shrinks thermally or squeezes out the water layer after shrinking (Figure 4a). Its advantages lie in its spatiotemporal controllability (millisecond response) and penetration regulation (suitable for deep-water environments). It has been applied to the fields of the light-driven lubrication switching of underwater robot joints and anti-adhesion coatings for minimally invasive surgical instruments.

Sun [43] et al. introduced AuNPs into the PNIPAAm hydrogel to prepare an anisotropic hydrogel actuator similar to an earthworm, and its lubrication properties were studied. Due to the good photothermal effect of AuNPs, the temperature of the hydrogel can be raised to 85 °C by irradiating it with a 445 nm laser for 30 s, and the volume can increase to 180% in less than 0.5 s. According to the change in volume, this hydrogel can crawl like an earthworm. In the laser irradiation area, the hydrogel became longer and thinner, and, due to the thermal contraction of the PNIPAAm hydrogel, its friction with the capillary was reduced. When the laser was moved, the shape and friction of the hydrogel in the previous area were restored, while the hydrogel in the newly irradiated area became longer and thinner. This swelling/thinning behavior allowed the hydrogel to crawl like an earthworm. The movement direction of the hydrogel can also be adjusted by changing the direction of the laser scanning. Inspired by muscle, Zhu [44] used similar materials to prepare nanocomposite hydrogels. Under the action of light, this PNIPAAm hydrogel containing AuNPs deformed rapidly and increased the friction with the hydrophobic substrate, achieving multifunctional gaits such as directionally controllable crawling, walking, and turning. One of the keys to hydrogel crawling is converting deformation into friction for directional motion. When the temperature was increased, the friction coefficient of PNIPAAm hydrogel and composite hydrogel containing AuNPs increased due to the change in hydrophobicity. However, when the light was turned on or off, the friction coefficient of the composite hydrogel can increase or decrease, but the PNIPAAm hydrogel did not change significantly. This muscle-like hydrogel provides new ideas for the development of soft robots.

In addition to controlling the bending of hydrogels via photothermal stimulation, it is also possible to control the contraction of hydrogels for transportation purposes. Inspired by the esophageal feeding mechanism, Liu et al. [45] prepared a PSPMA (poly(3-sulfopropyl methacrylate potassium) brush-grafted poly (AAc-NIPAM) hydrogel tubular actuator (HT-g-PSPMA) to propel the ball through photothermal-induced cavity deformation and lubrication (Figure 4b). The friction of HT-g-PSPMA increased from ~0.02 N at 25 °C to ~0.07 N at 50 °C. The increase in friction with increasing temperature was due to the volume contraction of the hydrogel. When Fe_3_O_4_ NPs are introduced, the contraction of the cavity can be achieved via photothermal control. By irradiating with near-infrared light, the hydrogel tube locally contracts, generating enough pressure to push the ball forward, which was similar to the mechanism whereby humans swallow food. Inspired by gastrointestinal peristalsis, Zhang [46] prepared a double-layer tubular hydrogel actuator (DLTHAs). The outer layer of hydrogel (PNIPAM/AG/CNTs) shrank and produced a water layer under the action of near-infrared light, and the super-hydrophilic inner layer formed a lubricating layer. Under the synergistic effect of the outer and inner layers, the viscous liquid can be pushed. Compared with temperature, photothermal regulation was faster and more convenient and was not limited by the environmental space.

Liu [47] prepared a PNIPAM nanogel (MTNGs, Microgel-based Tough Nanocomposite Hydrogel) containing Fe_3_O_4_ as a lubricant, and its tribological properties could be adjusted by temperature, magnetic field, and near infrared. Compared with water, the friction coefficient of the MTNGs suspension decreased from 0.087 to below 0.05. The reasons for the decrease in the friction coefficient were threefold: (1) the hydration layer around the PNIPAM chain was difficult to squeeze out; (2) increasing viscosity can form a stable elastic fluid film; (3) the nanogel can be used as a rolling bearing, and the friction coefficient of rolling friction was low. As the temperature increased, the hydration layer was destroyed, the MTNGs changed from hydrophilic to hydrophobic, and the friction coefficient also increased from ~0.026 to ~0.17. The magnetic field can also increased the friction coefficient by destroying the stability of the colloidal dispersion system. At the same time, Fe_3_O_4_ can absorb near-infrared light and convert it into heat energy. Under near-infrared light irradiation, the friction coefficient of the composite material increased. Chen [48,49] prepared [P(NIPAM-co-AA)@Au] near-infrared photothermal responsive microgels (PTMGs), which consisted of a thermosensitive gel shell and a near-infrared photothermal core, and its lubrication effect can be regulated by near-infrared light. Below the LCST, PTMGs were hydrophilic and form a hydration layer, which was in a low-friction state. When irradiated with near-infrared light and the system temperature was higher than the LCST, the PTMGs became hydrophobic, the hydration layer was destroyed, and it was in a high-friction state. By replacing Au with Fe_3_O_4_, the friction coefficient of the prepared Fe_3_O_4_ microgel can also be adjusted by near-infrared light, increasing from 0.17 at 25 °C to 0.55 at 40 °C (Figure 4c).

Wu [50] introduced MXene (two-dimensional carbides and nitrides) into a PNIPAm/PAMPS (poly-N-isopropylacrylamide/poly-2-acrylamido-2-methyl-1-propanesulfonic acid) double-network hydrogel and achieved the photothermal regulation of the friction of the hydrogel (Figure 4d). The introduction of chitosan significantly improved the dispersibility of MXene in the hydrogel. At 25 °C, which was lower than the LCST, the hydrogel exhibited a low friction coefficient due to the presence of a hydration layer on the surface. When irradiated with near-infrared light, the temperature of the hydrogel rapidly rose to above the LCST due to the good photothermal effect of MXene. At this time, the hydration layer was destroyed, causing the friction coefficient of the hydrogel to rise rapidly. The friction coefficient can also be switched back and forth between high and low through the “on–off” switching of the near-infrared light. This provides new bases for the construction of functional MXene hydrogels for smart lubrication, as well as the design of interface sensing, control transmission, and flexible robotic arms.

### 2.4. Photoresponsive Lubricating Hydrogels

There are many light signals in seawater, mainly including natural light sources and artificial light sources. Natural light sources are centered on solar radiation (mainly 400–700 nm visible light, of which blue-green light penetrates the deepest) and bioluminescence (such as the 480 nm blue light produced by plankton), which drive photosynthesis, biological behaviors (such as courtship and enemy avoidance), and chemical processes (such as UV-induced DNA repair). Artificial light sources include underwater operation lights (such as 450–550 nm LEDs) and monitoring lasers (such as 532 nm LiDAR (Light Detection and Ranging)), which may interfere with biological rhythms. These light signals change with depth stratification—the surface is dominated by sunlight, the middle layer retains blue-green light, and only bioluminescence remains in the deep layer. Their distribution directly affects ocean productivity, species distribution and environmental monitoring accuracy.

Photoresponsive lubricating hydrogels can dynamically regulate lubrication properties through light stimulation and exhibit unique non-contact control advantages in water environments. This type of gel is usually constructed by combining photosensitive components (such as photoisomerizable molecules such as azobenzene and spiropyran) with hydrophilic polymer networks. Its lubrication mechanism is based on light-induced molecular conformational changes: under light of a specific wavelength, the photosensitive group undergoes reversible isomerization (such as cis–trans conversion or a ring-opening reaction), triggering a rapid change in the hydrophilicity, charge distribution or swelling state of the polymer network (the response time can be seconds), thereby regulating the thickness of the interfacial hydration layer and the friction coefficient (adjustable range 0.02–0.5). Compared with other types of stimulus responses, the photoresponse has temporal and spatial precision and remote controllability, and it is particularly suitable for application scenarios in deep water environments or closed systems, such as light-controlled lubrication switches for underwater machinery and anti-adhesion coatings for minimally invasive surgical instruments.

The mechanism of photoresponsive lubrication is similar to shear force response, that is, under the action of light, the supramolecular non-covalent network in the hydrogel disassembles and forms a lubricating layer. By modifying irradiation parameters such as irradiation power, density, and cycle, the irradiation dose can be precisely adjusted. Unlike photothermal responses, the photoresponsiveness here is mostly a response to ultraviolet visible light, and the response light wavelength is mostly below 780 nm.

Wang [51] composited α-CD/PEG and a competitive agent, AzoPB, into a PVA and PAAm framework to prepare a semi-convertible supramolecular lubricating hydrogel (PSCH) with photoresponsive lubrication abilities. Under the action of visible light, the competitive photoresponsive agent, trans-AzoPB, destroyed the interaction between α-CD and PEG, forming a sol layer on the surface of the hydrogel, thereby reducing the friction coefficient from 0.0144 ± 0.0035 to 0.0045 ± 0.0022. When treated with UV light, the friction coefficient can be restored to ~0.015 due to the conversion of the competing agent AzoPB from trans to cis; the α-CD and PEG interaction was restored and the sol layer disappeared, and this process was reversible.

Yu [52] prepared a carboxylate- and sulfonate-rich polyanionic hydrogel (CS hydrogel) that uses iron ions to improve mechanical properties while also using iron ions as a reaction source (CS-Fe hydrogel). The original CS hydrogel had a low friction coefficient of about 0.05, and, after Fe^3+^ cross-linking, the friction coefficient increased to about 1.0; however, its mechanical properties were significantly improved. After ultraviolet irradiation, Fe^3+^ was reduced to Fe^2+^, and the upper hard hydrogel layer was transformed into a loose hydrogel layer, which greatly reduced the friction coefficient to about 0.02. This CS-Fe hydrogel had good biocompatibility and could protect fibroblasts/chondrocytes from inflammation, making it a potential candidate material for cartilage engineering.

### 2.5. pH-Responsive Lubricating Hydrogel

**Figure 5:**
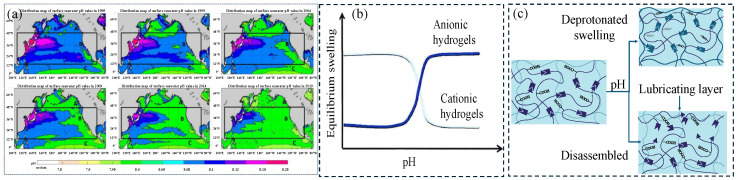
The schematics of pH-responsive lubricating hydrogel. (**a**) Spatial distribution of surface seawater pH values every five years, redrawn from [53]. (**b**) Swelling/deswelling degree of cationic and anionic hydrogels under different pH conditions. (**c**) pH-responsive lubrication mechanism: swelling caused by deprotonation of polymer chains (top); or formation of lubricating layer after chain disassembly or bond breaking (bottom).

Changes in the pH value in water are affected by both natural processes and human activities, showing multi-scale variation characteristics. Seasonal changes are manifested in the consumption of CO_2_ and increased alkalinity in the upper seawater in summer due to warming and enhanced photosynthesis, while the opposite is true in winter. The diurnal variation is particularly significant in summer. During the day, photosynthesis dominates, causing the pH to peak in the afternoon, while respiration causes the pH to drop at night. In winter, the variation decreases due to reduced biological activity. Additionally, regional differences show that areas with high dissolved oxygen levels generally have higher pH values. The impact of ocean acidification caused by human factors is more far-reaching. If carbon emissions continue, its rate will far exceed natural changes. By collating data from 1995 to 2019, a spatial distribution map of surface seawater pH was drawn every five years (Figure 5a) [53].

A pH-responsive lubricating hydrogel can dynamically adjust its lubrication properties through changes in environmental pH; it exhibits excellent stimulus-responsive properties in an aqueous environment. This type of gel is usually composed of a polymer network (such as polyacrylic acid, chitosan or poly(dimethylaminoethyl methacrylate)) containing pH-sensitive groups (such as carboxyl, amino or sulfonic acid groups). Its lubrication mechanism mainly relies on pH-induced molecular conformational changes: under specific pH conditions, the ionized groups on the polymer chain undergo protonation/deprotonation transitions, causing reversible changes in the network charge state, swelling degree and surface hydration properties, thereby regulating the interfacial friction behavior (the friction coefficient can be adjusted in the range of 0.01–0.5). In freshwater environments, this type of gel mainly relies on pH-induced charge changes to regulate lubrication properties. When the pH deviates from the isoelectric point, the ionization of the carboxyl/amino groups on the polymer chain causes the gel to swell and enhance surface hydration, significantly reducing the friction coefficient (to the order of 0.01), making it suitable for precise control systems such as microfluidic valves. In high-salinity marine environments, salt ions shield the charge effect of the polyelectrolyte network, resulting in a weakening of traditional pH responsiveness. For this purpose, salt-resistant hydrogels (such as sulfonic-acid-modified or zwitterionic polymers) can maintain controllable lubrication (friction coefficient 0.05–0.3) in a wide pH range (7–9) by enhancing ion pair stability and osmotic pressure regulation abilities, and they are used in ship antifouling coatings or deep-sea equipment interfaces. The performance difference between the two environments is mainly due to the interference of ionic strength in the Donnan equilibrium (Note: Donnan equilibrium refers to a state where, due to the uneven distribution of non-diffusible large molecules (e.g., large ions in a polymer electrolyte solution) across a semi-permeable membrane, the distribution of small diffusible molecules or ions on either side of the membrane becomes unequal while reaching a balance, with the product of the concentrations of cations and anions on each side being equal). The current research optimizes environmental adaptability by constructing hydrophobic microdomains (reducing salt sensitivity) and dynamic covalent networks (improving mechanical robustness) to provide customized solutions for different water scenarios.

Hydrogels containing dynamic covalent bonds (such as borate bonds, acylhydrazone bonds, imine bonds, etc.) will undergo a gel–sol transition as the pH of the external environment changes. pH-responsive hydrogels tend to respond to specific pH levels and exhibit swelling or deswelling behaviors. pH-responsive polymers usually contain side groups, generally acidic groups such as carboxylic acids or basic groups such as ammonium salts, which enable them to absorb or release protons in response to pH changes in the environment. When the surrounding pH is higher than the pKa value of the acidic side groups on the polymer chain, the acidic groups will lose protons to form negatively charged polymer chains that interact with the positively charged flowing solution. This will increase the electrostatic repulsion between the negatively charged chains, causing the hydrogel network to swell. When the surrounding pH is lower than the pKa value of the basic side groups on the polymer chain, the basic groups will accept protons to form positively charged fixed polymer chains that interact with the negatively charged flowing solution. This will increase the electrostatic repulsion between the positively charged chains, causing the hydrogel network to swell, as shown in Figure 5b.

The lubrication mechanism of pH-responsive hydrogels is mostly based on the swelling or shrinking of the hydrogel network caused by deprotonation or protonation (Figure 5c, top). If the polymer chain contains weak acid or weak base groups (such as carboxyl or amine groups), swelling will reduce the friction coefficient of the hydrogel due to the enhanced hydration effect; shrinkage will increase the friction coefficient. Another lubrication mechanism involves the disassembly or bond breaking of pH-responsive polymer chains to form a lubricating layer, thereby reducing the friction coefficient.

Kang [54] prepared a carbon-dots-enhanced pH responsive lubricating hydrogel (CPLH) and studied its pH responsive lubricity. The CPLH was composed of PVA-borax dynamic covalent bonds and PAAm covalent networks. The pH responsiveness of the borate bonds endowed the hydrogel with the ability to regulate lubrication. When the pH value was less than 6.0, the friction coefficient of CPLH could be reduced to 0.041 ± 0.003, which was attributed to the sol layer formed on the surface of the hydrogel. In addition, due to the introduction of blue-light-emitting CDs, the formed sol layer can be observed using fluorescence microscopy. When the pH was greater than 8.5, its friction coefficient could be restored to 0.069 ± 0.011, and this reversible tribological process can be undergone many times. The introduction of CDs not only improved the lubrication ability of the hydrogel but also improved its mechanical properties. This study provides a promising basis for designing pH-responsive soft actuators or soft robots, and future work will develop more durable and sensitive lubricating hydrogels.

Inspired by the lubricity of fish skin, Wu [55] prepared a pH- and temperature-responsive PNIPAAm (poly(N-isopropylacrylamide)-NaMA hydrogel and investigated its lubrication properties. This hydrogel had an ultra-low friction coefficient, dual stimulus responsiveness, and dynamic lubrication regulation. The swelling of the polymer chains and the water stored in the polymer network gave the hydrogel an ultra-low friction coefficient of 0.05. When pH = 2, the PNaMA (pH-responsive component) shrank and collapsed, resulting in a reduction in the water film and an increase in the friction coefficient to 0.16. When the temperature rose to 32 °C (pH = 2), the friction coefficient further increased to about 1.0. The reason for the increase in the friction coefficient may be due to the double collapse of PNaMA and PNIPAAm (the temperature-responsive component), which led to the further destruction of the water film, so the friction coefficient increased sharply. It is worth noting that similar results were obtained when DMAEMA was used instead of NaMA. Ma [56] prepared an ordered hydrogel nanofiber array composite surface and studied its lubricity. The hydrogel nanofiber composite surface was composed of PAA (polyacrylic acid) hydrogel nanofibers enclosed in an anodic aluminum oxide (AAO) template (used as a nanocolumnar porous reservoir). The PAA hydrogel nanofibers had excellent lubricity, while the AAO template was able to withstand high loads. When the surface layer was destroyed, the hydrogel could be be released to regulate lubrication. Under a high load (40 N), it exhibited an ultra-low friction coefficient (0.007), while, under a low load (3 N), the hydrogel exhibited a high friction coefficient (0.1). The reason for the low friction coefficient may be that the formation of the high-density viscous polymer gel destroyed the ordered gel array structure. The friction coefficient of the composite surface can also be adjusted by the pH solution. In a weakly acidic medium (pH = 3, lower than the pKa of PAA, deswelling state), the composite surface exhibited high friction; in a weakly alkaline medium (pH = 10, higher than the pKa of PAA, swelling state), the composite surface exhibited superlubricity. The friction coefficient of the composite surface was reversible and fast-responsive.

In addition to traditional pH-responsive lubricating hydrogels, there is also a special type of lubricating hydrogel that uses an alkali-induced strategy to form a soft and porous layer, resulting in a lower friction coefficient. Qu [57] used an alkali-induced network dissociation strategy to prepare a PAA-Fe (Polyacrylic Acid-Fe)/PAAm (polyacrylamide) double-layer hydrogel. After the alkali treatment, the top of the hydrogel softened and formed a porous structure, while the bottom remained solid. The friction coefficient of the hydrogel surface can be adjusted by the alkali treatment time. As the alkali treatment time increased from 30 s to 5 min, the friction coefficient decreased from 0.04 to 0.015. This lubrication mechanism can be summarized as follows. After processing, the surface became looser and more porous, and the porous structure was able to absorb a large amount of water and provide good lubrication; the bottom layer had sufficient load-bearing functions. Huang [58] used a similar method to form a soft hydrogel layer on the hard agar/PAMAAc (poly(acrylamide-co-acrylic acid))-Fe^3+^ hydrogel (PAMAAc: PAAm/PAAc double network hydrogel) to prepare a double-layer-structure composite hydrogel. The agar/PAMAAc hydrogel on the upper layer provided good lubricity, while the agar/PAMAAc-Fe^3+^ hydrogel layer on the lower layer provided load-bearing capacity. After 20 min of alkali treatment, the friction coefficient was reduced to 0.024 and remained at a low level (no more than 0.05) after 50,000 test cycles.

Ahmed [59] studied the changes in the tribology of the PCDME (poly(N-(carboxymethyl)-N, N-dimethyl-2 (methacryloyloxy) ethanaminium) hydrogel in different ionic solutions and pH environments. When the ionic strength was controlled at 0.1 M, the friction coefficient remained almost unchanged in the pH range of 1.9 to 6.63, but, when the pH value exceeded 8.5 (the isoelectric point was 8.3), the friction coefficient began to decrease due to electrostatic repulsion. Zhang [60] prepared poly(NIPAm (N-isopropylacrylamide-co-acrylic acid)-AA) microgels. The higher charge density made the microgels more hydrating. At a high pH, the friction coefficient was low due to the high hydration caused by deprotonation. At a low pH, the microgels were easily sheared off due to the weak adsorption capacity, and the exposed PMETAC polymer brushes made the friction coefficient lower. Ma [61] prepared a PAA double-sided nanohydrogel brush structure distributed on both sides of the membrane. The composite membrane can exhibit different friction states, depending on different pH combinations. The reason for the different friction states was the swelling or shrinkage of PAA in alkaline or acidic media. In acidic media with pH = 2, the composite membrane showed a high friction coefficient of about 0.3~0.4 due to the high surface roughness (~172 nm) caused by the shrinkage of PAA; on the contrary, in alkaline media with pH = 12, PAA swelled and formed a uniform and smooth surface (~5.6 nm), which showed an ultra-low friction coefficient of less than 0.01. In an acidic medium with pH = 2, a gradual decrease in the friction coefficient can be observed by continuously adding an alkaline medium (pH = 12) to the interface; when an acidic medium was added, the friction coefficient increased, and the change in the friction state was reversible.

Zhang [62] prepared a multi-responsive composite surface made of poly(AA/SPMA, acrylic acid/sulfopropyl methacrylate) hydrogel fibers embedded in an anodic aluminum oxide (AAO) nanoporous substrate. Due to the excellent hydration ability of SPMA, its friction coefficient can reach 0.01. This hydrogel fiber composite surface can respond to pH, ions and surfactants. When the PAA hydrogel fibers were immersed in buffers of different pH values, their friction coefficient decreased from 0.40 to 0.007 with the increase in the pH value. The deprotonation of carboxylic acid groups caused the polymer chains to swell, thereby reducing the friction coefficient (except for poly (AA/SPMA0.5), whose large number of sulfonic acid groups put the system in a dissociated state and was not affected by pH value). As the concentration of NaCl in the buffer increased, the friction coefficient of the hydrogel fiber composite surface increased from 0.01 to 0.29 due to the collapse of the polymer chains. With the addition of Ca^2+^, Cu^2+^, and Fe^3+^ in the buffer, the friction coefficient increased from 0.01 to 0.20, 0.27, and 0.31. This can be explained by the chelation of carboxylic acids with metal ions, which neutralized the charge of the polymer chains and forced them to collapse, thereby increasing the friction coefficient. When immersed in a cationic surfactant (CTAB), the friction coefficient increased from 0.003 to 0.11; while when immersed in an anionic surfactant (SDS), the friction coefficient did not change significantly. The interaction between positive and negative charges reduced the repulsive force between the sliding interfaces, thereby increasing the friction coefficient.

Inspired by sea cucumbers and muscles, Hu [63] prepared a pH- and temperature-responsive graphene oxide hydrogel. Due to the internal microstructure reconstruction caused by pH and heat, the prepared hydrogel can rapidly change its mechanical properties and friction coefficient. The friction coefficient of the hydrogel increased from ~0.05 at pH = 2 to ~0.25 at pH = 10. When the temperature increased from room temperature to 75 °C, the friction coefficient increased from ~0.05 to ~0.16.

### 2.6. Chemical-Signal-Responsive Lubricating Hydrogels

Chemical signals in the aquatic environment include two main categories: natural secretions and anthropogenic pollutants. Natural signals include biological pheromones (such as fish sex pheromones regulating reproduction), metabolites (such as dimethyl sulfide released by phytoplankton affecting the climate), and nutrient gradients (such as nitrogen and phosphorus triggering algal blooms); anthropogenic signals include persistent organic pollutants, drug residues, and microplastic additives (such as phthalates). These chemical signals regulate ecosystems through intraspecies communication, interspecies interactions, and environmental stress responses, and their detection technology is widely used in ecological monitoring and restoration. Current research focuses on the analysis of chemical signal networks and the impact of climate change on their transmission efficiency.

Chemical-signal-responsive lubricating hydrogels can specifically recognize specific chemical factors in the environment and dynamically regulate interfacial friction behavior, showing unique molecular recognition and adaptive properties in water environments. This type of gel is usually constructed from functional polymers with chemical recognition sites (such as crown ether-modified polyelectrolytes, molecular imprinting networks, or enzyme-responsive matrices). Its lubrication mechanism is based on specific molecular interactions triggered by chemical signals. For example, when target chemicals (such as metal ions, biomolecules, or pH changes) are detected, the gel network undergoes controllable swelling/contraction through host–guest interactions, coordination bond reorganization, or enzyme-catalyzed reactions, thereby regulating the structure of the interfacial hydration layer or releasing boundary-lubricating molecules (friction coefficient adjustable range 0.01–0.5). By designing specific recognition groups (such as thiol, carboxyl, or bioreceptors) and dynamic cross-linking strategies (such as reversible covalent bonds), highly selective responses and rapid recovery can be achieved, which has important application value in environmental monitoring (such as heavy metal ion sensing), biomedicine (such as inflammation-responsive artificial joints), and underwater soft robots.

Chemical signals come from many sources, are widely distributed, and are very different from each other; therefore, the associated lubrication mechanism is relatively complex. For example, (1) chemical components may cause the covalent polymer network to shrink or swell, thereby changing the structure or adhesion of the hydrogel surface, which in turn causes responsive lubrication; (2) chemical components may also cause the responsive components in the system to decompose, assemble, or break bonds, forming a lubricating layer, which brings about changes in surface lubrication.

Inspired by the tongue’s ability to perceive astringency, in Ma and Khan’s work [64,65], the PAAm hydrogel was prepared to simulate the failure of lubrication when protein MP (mucoprotein) encountered polyphenol compounds (TA, tannic acid). The prepared hydrogel was flexible and has low friction when in water, but it became viscous after being treated with TA solution, with increased mechanical strength but increased friction. The mechanism may be that protein dehydration causes the gel network to shrink, thereby changing its own lubrication properties. Based on this mechanism, the team designed and developed a TA-containing glove for catching slimy fish.

Zhao [66] introduced phenylboronic acid into a double-network hydrogel (poly (AMPS-co-AAm)/PAA), giving the hydrogel glucose-responsive lubrication characteristics. The friction coefficient of the hydrogel changed with the change in the glucose concentration, with a maximum of 0.06 and finally decreasing to about 0.025. The change in the friction coefficient was attributed to the formation of the hydration layer and the binding ratio of glucose to APBA (3-Aminobenzeneboronic acid). Different binding modes led to different pore sizes in the hydrogel. When the binding ratio of glucose to APBA was 1:2, the pore size decreased, resulting in a decrease in the friction coefficient. In addition, the prepared hydrogel can also respond to solutions of different pH values because phenylboronic acid is also a pH-responsive group.

Huang [67] prepared a PTMAEMA/PSBVI (poly((trimethylamino) ethyl methacrylate chloride)/poly (sulfobetaine vinylimidazole)) interpenetrating network hydrogel and studied its lubricating and antibacterial properties. In deionized water, the friction coefficient of the PTMAEMA/PSBVI hydrogel was about 0.8, but, in 1.0 M NaCl solution, the friction coefficient was significantly reduced to about 0.1. This reduction can be attributed to the anti-electrolyte effect of the PSBVI hydrogel, which increased hydration. Due to the opposite swelling behavior of the salt response, different optical structures can be observed under the microscope, with the outer layer structure as a transparent layer and the inner layer structure as an opaque area. Interestingly, the bactericidal rate of this hydrogel against Staphylococcus epidermidis was over 80% and the bactericidal rate against Escherichia coli was over 90%. In addition, this hydrogel can release 96% of bacteria after washing with 1.0 M NaCl solution, which provides a new conceptual basis for the application of smart materials in medical and industrial fields.

Xiao [68] designed a sodium alginate (SA)/(3-(1-(4-vinylbenzyl)-1H-imidazol-3-ium-3-yl)propane-1-sulfonate (VBIPS)-co-2-hydroxyethyl acrylamide (HEAA) double-network hydrogel based on a special oligomer monomer VBIPS and explored its ion-responsive lubrication properties. When the hydrogel was immersed in water, its friction coefficient stabilized at ~0.3. After treatment with 1.0 M NaCl, its friction coefficient dropped to about 0.05. The mechanism of action was that, when NaCl enters the hydrogel, the counterions penetrated into the oligomer chains, destroying the electrostatic interaction, thereby promoting the interaction between the polymer and water, leading to the extension of the polymer network. The loose polymer network caused part of the solution to seep to the surface of the hydrogel under the action of external force, thereby reducing the friction coefficient. Hydrogels with different compositions showed different friction coefficients, and the friction coefficient decreased with the increase in polyVBIPS content. When the concentration of the NaCl solution was higher than 0.53 M, the friction coefficient stabilized at about 0.05. Different anions also affected the friction coefficient of the hydrogel. SO_4_^2−^ resulted in a higher friction coefficient (~0.53), while Br^−^ and NO^3−^ resulted in a lower friction coefficient (~0.034 and ~0.032, respectively).

Ions can regulate the decrease in friction or the increase in the friction coefficient, a process that mainly depends on the polymer network. Xiao [69] prepared a bactericidal-releasing smart hydrogel surface composed of poly(HEAA-co-METAC (methacryloxyethyltrimethyl ammonium chloride)) hydrogel grafted with PSBMA brushes. Through opposite volume and conformational changes, this hydrogel–brush hybrid coating can achieve smart bactericidal or bacterial release. The friction coefficient of the hydrophilic poly(HEAA-co-METAC) hydrogel in water was low, about 0.1, while the friction coefficient value in the NaCl solution rose rapidly to about 0.3. After adding the PSBMA-poly (HEAA-co-METAC) coating, the original friction coefficient ~0.15, increased to ~0.3 after immersion in NaCl. The change in the friction coefficient can be attributed to the polyelectrolyte effect of cationic polymers, i.e., they swelled in water and shrank in ionic media. The swelling led to a low friction coefficient, while shrinking led to a high friction coefficient.

### 2.7. Electric-Field-Responsive Lubricating Hydrogel

Seawater is an electrolyte solution with ion movement inside, which generates a weak electric field [70]. Human activities in the ocean, such as the operation of marine engineering, offshore wind farms and other facilities, also generate underwater electromagnetic fields. In the marine environment, metal pipelines generate corrosion electric fields due to preload and corrosion defects. Taking the 20# steel metal pipeline with ellipsoidal corrosion defects as an example, local elastic–plastic deformation often occurred at the defect, affecting the corrosion electric field characteristics of the metal pipeline. Different metal pipes were connected and sealed by applying a pre-tightening force on the flange surface. When there were corrosion defects, the geometric shapes and structures of the defective parts were different, which caused stress and strain to increase and elastic–plastic deformation to occur, thus generating corrosion electric fields [71]. Liu [72] used the finite element method to establish an electric field model of submarine-impressed current cathodic protection. The analysis showed that the anode current increased significantly with the increase in seawater conductivity (the seabed conductivity and depth had little effect), while the underwater electric field peak decreased with the increase in seawater conductivity, seabed conductivity, and depth.

**Figure 6:**
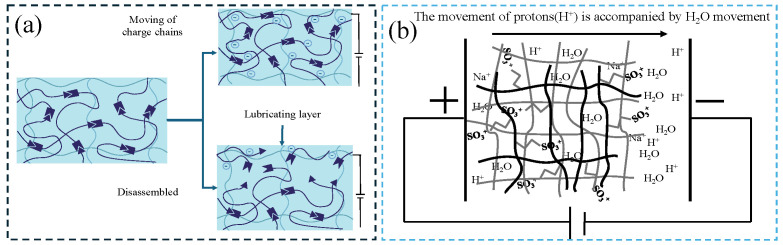
Schematics of chemical signal-responsive & Electric field responsive lubricating hydrogel. (**a**) Electro-Responsive lubrication mechanism: the directional movement of charged chains causes the change of surface charge of the hydrogel (top); Electro-Responsive disassembly forms a lubricating layer (bottom). (**b**) DN gel friction change mechanism (protons move to the cathode side under the action of electricity) and optical photos of the robot walking in sequence, figure adapted from Selvamuthu et al. [74,75] under BY-NC-ND 4.0 license.

Electro-responsive hydrogels are hydrogels that can respond to changes in electric fields and change their properties (contraction, expansion, bending, etc.) accordingly, generating friction properties. The mechanism is based on the directional migration of hydrophilic groups or charged molecules inside the hydrogel under the action of an electric field, thereby changing the thickness of the surface hydration layer or the arrangement of lubricating molecules, and achieving the dynamic regulation of the friction coefficient. This type of hydrogel is usually composed of a polyelectrolyte network (such as polyacrylic acid, chitosan) or a conductive polymer (such as polyaniline), with a high water content (> 80%) and fast responsiveness (seconds). In an aqueous environment, the electric field reversibly induces a hydrophilic–hydrophobic transition or ion release on the gel surface, forming a boundary lubrication layer with low shear strength, and the friction coefficient can be reduced to the order of 0.01, for example. When electric-field-responsive lubricating hydrogels are composed of polymers (or cross-linked polymer networks) with ionizable groups on the molecular chain, under the action of an electric field, the counterions of these charged groups migrate, causing changes in the ion concentration of the polymer chain (or inside and outside the gel network), which in turn triggers a phase transition, thereby achieving the controllable adjustment of the lubrication performance. There are two mechanisms of electro-responsive lubrication. One is the directional movement of charged chain segments (Figure 6a, top), which changes the surface charge of the hydrogel. If the surface charge is the same as the charge of the friction pair, the electrostatic repulsion effect will reduce friction. Conversely, the electrostatic attraction effect will increase friction. The other mechanism is the disassembly of the electro-responsive chain segments. The formed lubricating layer will reduce the friction coefficient of the hydrogel surface (Figure 6a, bottom).

Inspired by the ability of mucosa to secrete mucus, Kang [73] prepared a SF-PAAm/PVA electro-responsive lubricating supramolecular-covalent hydrogel (electro-responsive lubricating supramolecular covalent hydrogel, ESCH). Under the action of an electric field (30 V), the gel-state SF supramolecular hydrogel disassembled, and ESCH underwent a partial gel–sol transition, forming a sol layer on the surface, and the friction coefficient of the hydrogel decreased from 0.115 ± 0.005 to 0.062 ± 0.003. Moreover, when the positive and negative poles of the electric field are switched, the friction coefficient can be restored to about 0.115, and this process can be repeated many times, achieving reversible lubrication behavior. This property makes it possible to use the material as a driving material for soft robots, achieving robot movement and control by controlling the electric field. This also gives it broad application prospects in condition where the dynamic adjustment of lubricity is required, such as mechanical seals, bearings, and other fields.

Takata [74] studied the effect of different voltages on the friction coefficient of the PNaAMPS (poly(sodium-2-acrylamido-2-methyl-1-propanesulfonate)) hydrogel. The experimental results showed that the friction coefficient increases significantly with the increase in voltage, and, when the electric field was turned off, the friction coefficient returned to its original state. The increase in the friction coefficient may be due to the attraction between the charges on the PNaAMPS hydrogel and the surface charges caused by the electric field. The voltage induced positive charges on the substrate surface, attracting the SO^3−^ groups of the polyelectrolyte gel, thereby increasing the friction coefficient. Subsequently, the hydrogel was immersed in surfactants to investigate the lubrication of the hydrogel in different surfactants. When the electric field was turned on, the friction torque dropped to about 0.2 mN·m (anionic surfactant SDS); when the electric field was turned off, the friction torque returned to its original value. The reason for the decrease in friction torque may be that there was a lubricating layer between the PAAm hydrogel and the substrate, which was brought to the surface of the hydrogel by the internal anionic surfactant under the action of the electric field. However, for the cationic surfactant (DTAB), the friction torque of the hydrogel increased under the action of the electric field, because the cationic surfactant near the interface moved into the hydrogel, thereby increasing the friction torque.

Selvamuthu [75] placed the PNaAMPS/PDMAAm double-network hydrogel (DN gel) on the feet of a walking robot. Under the action of an electric field (Figure 6b), the friction of the hydrogel can be controlled, thereby controlling the directional movement of the robot. The mechanism of the change in the friction coefficient of the hydrogel was due to the protons (H) in the solution inside the hydrogel. By increasing the voltage, these protons were moved to the cathode side. The movement of the protons caused an osmotic imbalance between the anode and the cathode. Therefore, water molecules also moved to the cathode side, thereby forming a lubricating layer between the polymer network and the cathode electrode, which can reduce the friction coefficient from 0.04~0.07 to 0.02. Based on the reduction in the friction coefficient, the robot can be controlled to move like an inchworm.

In addition to applying an electric field directly to the hydrogel, the friction condition can also be changed by changing the charged state of the friction pair. Wada [76] studied the lubrication of the friction pair on the PAMPS/PDMAAm double-network hydrogel under different voltages and charged states. When the measuring ball was the cathode, the voltage was 0.0~3.0 V, and the friction coefficient of the hydrogel decreased; when the voltage was 3.0~5.0 V, the friction coefficient increased. When the measuring ball was the anode, the friction coefficient increased with the increase in the voltage. The change in the friction coefficient was attributed to the change in the water film under the electric field. An increase in the thickness of the water film caused the friction coefficient to decrease; conversely, a decrease in the thickness of the water film or its destruction led the friction coefficient to increase.

## 3. Water-Based Lubricant Composites Containing Hydrogels

Hydrogel lubricants are currently applied primarily in closed environments, such as human joints, where the lubricating medium is strictly confined and does not exchange substances with the external environment. This containment prevents the dissipation of the lubricant, thereby ensuring the long-term lubrication performance of the friction pairs. In contrast, certain engineering applications involve open lubrication systems. For example, in water-lubricated bearings, lubrication often relies on drawing water from a surrounding source or immersing the bearings directly in water. In such open systems, the direct application of hydrogel lubricants can lead to their dilution or being washed away.

To address these challenges, the hydration lubrication performance of hydrogels can be enhanced by incorporating them into the composite materials used in friction pairs. Using hydrogel particles as additives offers a theoretically viable approach to improving lubrication in aqueous environments. However, hydrogels tend to weaken mechanically after swelling, and their dimensional changes can disrupt the working gap between friction pairs. These limitations make them difficult to apply directly in heavy-load engineering contexts.

Common friction-pair materials used in water-based lubrication include ultra-high-molecular-weight polyethylene (UHMWPE) and polyurethane (PU). In recent years, research has increasingly focused on developing new strategies for hydrogel-containing composites, using these polymers to overcome the limitations of traditional hydrogel applications.

### 3.1. Applying Double- or Multiple-Networked Hydrogels

Studies have found that conventional single-network hydrogels are insufficient to meet the needs of engineering applications. For example, Wang et al. used single-network polyacrylamide (PAAm) hydrogel microspheres blended with UHMWPE and then prepared PAAm microsphere/UHMWPE composites via extrusion molding. Although the lubricity and wear resistance of the composites were improved, the addition of the PAAm hydrogel caused a considerable increase in the water absorption and swelling rate of the composites, as well as a decrease in the mechanical properties. This phenomenon became more obvious with the increase in PAAm hydrogel content [77].

Therefore, double-network or multi-network hydrogels have been prepared to construct composite materials. Huang et al. constructed a double-network hydrogel composed of PAAm and dopamine (PDA) and incorporated it into a rigid polyurethane foam (RPUF) through a simple coating–curing process. This significantly enhanced the flame-retardant performance of RPUF while maintaining its original mechanical properties and structural integrity [78]. Liao et al. used the freeze–thaw method for the in-situ formation of a polyvinyl alcohol (PVA) hydrogel inside melamine formaldehyde (MF) foam to prepare a PVA/MF composite with an interpenetrating double-network structure, which significantly improved the mechanical properties and water resistance [79]. Ge et al. proposed a strategy for 3D printing hydrogel–polymer composite structures, using photoinitiator TPO nanoparticles to initiate the partial polymerization of an acrylamide-PEGDA (AP) hydrogel, achieving covalent bonding between the AP hydrogel and a variety of UV-curable polymers. This study demonstrated the good compatibility and wide application potential of hydrogels and traditional polymers in three-dimensional composite structures [80]. Mu et al. constructed a polymer–hydrogel composite material with a low friction coefficient and strong durability by filling charged polymer chains into a porous hydrogel. The design mimicked the lubrication mechanism of synovial joints, where the porous structure was used to store mobile polymer chains, while charged polymer chains (such as sodium alginate) were able to form a stable hydrated layer at the interface. The hydration layer can maintain fluidity under pressure and shear due to the strong hydration between the polymer chains and water molecules, thereby providing continuous interfacial lubrication during sliding, effectively reducing friction and delaying surface damage [81].

**Figure 7:**
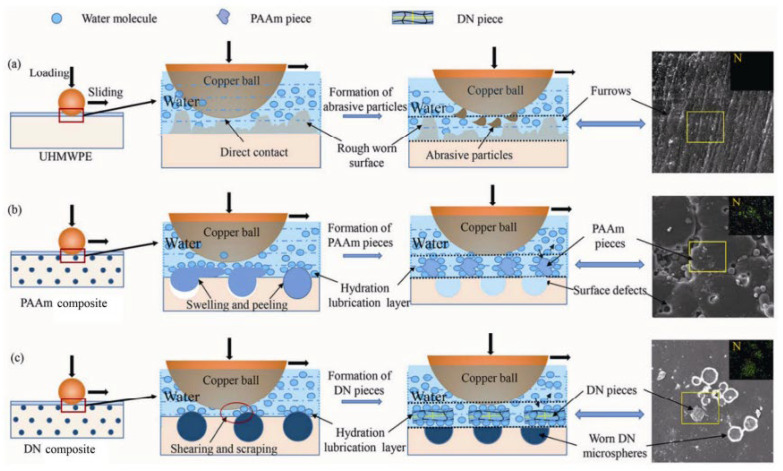
Schematic illustration of friction behavior in the rubbing process of the fracture pair: (**a**) UHMWPE, (**b**) PAAm microsphere/UHMWPE composite, and (**c**) DN microsphere//UHMWPE composite, figure adapted from Wang et al. [82] under CC BY license.

In order to solve the problem of the poor dimensional stability of the above-mentioned single-network hydrogel after swelling, Wang et al. prepared PAAm/urea-formaldehyde resin (UF) double-network (DN) hydrogel microspheres by adopting a reaction method of free radical polymerization/condensation polymerization in parallel. They prepared 75DN microsphere/UHMWPE composites by melt blending and extrusion molding, and the composites demonstrated improved stability and tribological properties [82]. As shown in Figure 7, the design of the soft/hard DN double-network composite structure effectively solved the problem of the swelling and shedding of hydrogel microspheres, ensuring that the composite disc can contact the wear pair with a smoother surface during the friction process. The presence of the UF hard network can ensure that the DN microspheres had good dimensional stability and high mechanical strength in the water environment. The DN microspheres on the friction surface of the composite disk can be gradually worn away in a layered form along with the wear of the UHMWPE matrix material, leaving many worn DN microspheres on the wear surface as supply points for hydrated lubricants. Under the action of shear force, the worn-away layered DN hydrogel were sheared and broken into many small fragments. These fragments were filled between the two wear surfaces, forming a hydrated lubricating layer composed of DN hydrogel fragments. The PAAm network in the DN hydrogel fragments had good hydrated lubrication and can provide lower friction resistance. Therefore, under the test conditions, the 50DN and 75DN microsphere composites had good hydrated lubrication, load-bearing performance and stability.

### 3.2. Addition of Reinforced Agents

Adding additives with good mechanical properties to the hydrogel-containing composite is another effective strategy. These reinforcing agents include graphene oxide (GO), montmorillonite, fiber, cellulose nanocrystals (CNC), etc. The abundant hydroxyl groups on the surface of the hydrogel can form hydrogen bonds, π–π stacking, bonding, etc., with these reinforcing agents, thereby ensuring the compatibility of the matrix. For example, Zhao et al. filled the hydrogel made via the copolymerization of polyethylene glycol (PEG) and polyacrylic acid (PAA) into the polyurethane (PU) foam, introducing graphene oxide and hexagonal boron nitride (h-BN) to improve its thermal conductivity and electrical insulation properties; they obtained a composite material with flexibility, thermal conductivity and vibration resistance. During the phase-change process, this material can enhance interfacial heat conduction, reduce the temperature rise caused by vibration, and improve the buffering capacity against external impact [83]. Farazin et al. prepared a composition of nanocomposites (0 wt.%, 5 wt.%, 10 wt.%, 15 wt.%, and 20 wt.% of Ag_2_O/SiO_2_) and found that the mechanical properties and the healing properties of the wound dressing increased significantly with the increase in Ag_2_O/SiO_2_ [84]. Li et al. developed a strategy for synthesizing CNC gels with tunable mechanics. Hydrogels (complex moduli: 160–32,000 Pa) and aerogels (Young’s moduli: 0.114–3.98 MPa) were produced by sequential freeze–thaw/hydrothermal treatments. The enhanced stability, low thermal conductivity (0.030 W/m K), and high-temperature resilience can expand their potential applications [85].

In response to the problem of the poor load-bearing capacity of hydrogel composites, the research team of Wuhan University of Technology conducted in-depth research and developed a series of load-bearing hydrogel/stiff polymer composites [86,87]. In the first composite system [86], the PAAm hydrogel layer was grafted on the surface of graphene oxide (GO) via in situ free radical polymerization to prepare PAAm-GO sheets. Since the PAAm-GO sheets had both the hydration lubrication ability of PAAm and the 2D shape effect of GO, the lubricating layer composed of PAAm-GO and its fragments can significantly reduce the occurrence of friction and wear behavior of the composite material. Under a load of 50 N, the PAAm-GO/UHMWPE composite effectively avoided the occurrence of local damage, improved the ultimate load-bearing capacity of the composite material, and had good load-bearing lubrication performance. In the second system [87], multi-network hydrogels were prepared using polyvinyl alcohol (PVA), chitosan (CS), sodium alginate (SA), etc., and montmorillonite (MMT) was introduced as a reinforcing agent. The results showed that montmorillonite reinforcement can effectively improve the load-bearing and lubrication properties of the material. The underlying mechanism was that polyvinyl alcohol, chitosan, and sodium alginate were entangled with each other to form a multiple-networked structure in the hydrogel. After using montmorillonite to enhance the load-bearing capacity of the hydrogel, the -NH group of CS was able to form a hydrogen bond group with the -OH group of MMT. Therefore, the hydrogel connected small-sized montmorillonites along the two-dimensional direction to form a large-sized sheet structure. Furthermore, as a reinforcing agent, montmorillonite merged with the CS chain to form a stronger planar structure. This transformed the general network structure of the original hydrogel into a porous structure and enhanced the load-bearing capacity of the hydrogel, thus giving the MMT-hydrogel/TPU excellent friction properties and wear resistance.

**Figure 8:**
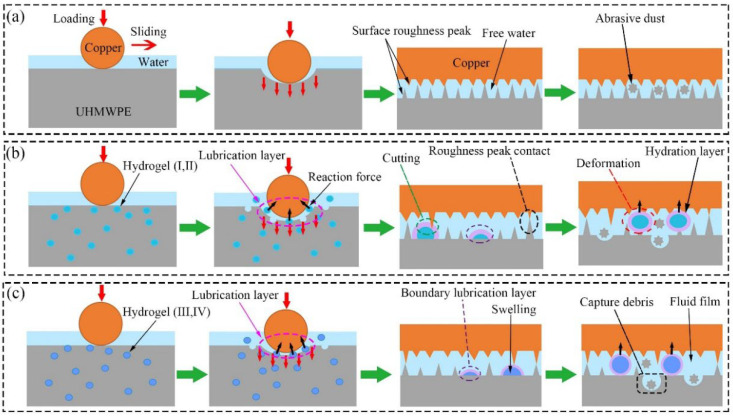
Schematics of the wear and lubrication mechanisms and processes of the ANF-reinforced hydrogel/UHMWPE composites, figure adapted from Li et al. [88] under CC BY license. (**a**) the control group, UHMWPE; (**b**) the composites with hydrogel I, II; (c) the composites with hydrogel III, IV. The thin red arrows present the pressure generated from copper ball; the short black arrows indicate the carried force.

Nanofibers are an effective additive. Aramid fiber (ANF) was introduced into PVA, CS, and SA multi-networked hydrogels, and the hydrogel/UHMWPE composite was prepared via hot pressing [88]. The results showed that the introduction of ANFs led to an increase in the cross-linking degree of the hydrogel, enhanced the rigidity of the hydrogel molecular network, and thus improved the fracture strength of the hydrogel. Compared with the hydrogels without ANFs (hydrogel I, II), the introduction of the high-rigidity hydrogels (hydrogel III, IV) further improved the surface hardness of UHMWPE, which made the surface of the composite material more resistant to damage from hard and rough peaks under load and reduced the wear rate. The hydrogel containing ANFs had better load-bearing capacity and was able to effectively separate the friction interface during friction. By adjusting the content of ANFs, a balance between hydration and the load-bearing performance was achieved, which improved the friction coefficient and wear rate of the composite material with the working mechanisms, as shown in Figure 8.

### 3.3. Self-Repairing Hydrogels Working Under Dynamic Conditions and Fast Speeds

The self-healing property of hydrogels mainly comes from the reversible cross-linking of polymer networks. According to the different cross-linking methods, the self-healing mechanism can also be divided into physical and chemical effects. Hydrogen bonds, electrostatic interactions, hydrophobic associations, and dynamic non-covalent bonds formed by host–guest complexation are all effective means of achieving the self-healing properties of hydrogels [89]. In addition, hydrogels cross-linked by certain dynamically reversible covalent bonds can also achieve self-healing effects. The reversible covalent bonds used to prepare self-healing hydrogels mainly include acylhydrazone bonds, imine bonds, borate bonds, disulfide bonds, diselenide bonds, Diels–Alder reversible bonds, thioester bonds, etc. Since hydrogen bonds are sensitive to pH and temperature, it is necessary to control pH and temperature to achieve the formation of hydrogen bonds and the self-healing of hydrogels [90]. The self-healing ability of hydrogels with electrostatic interactions depends on dynamic reversible charge attraction and dissociation. Their self-healing process requires maintaining specific external conditions such as the ionization state of positively and negatively charged groups in the hydrogel, the ion concentration in the environment, and the appropriate temperature to achieve network repair [91]. The self-healing ability of hydrophobic-associating hydrogels can originate from the dynamic dissociation and recombination of hydrophobic groups. It is necessary to regulate external conditions such as the temperature, mechanical contact, and solvent environment to achieve the efficient and reversible repair of the hydrogel cross-linked network [92]. In addition to factors such as temperature, pH, ionic strength, and light, free competing molecules on the interface will also affect the self-healing ability of host–guest complex hydrogels. Interestedly, Zhou et al. [93] reported a reusable (self-repairing) soft electronic skin tattoo sensor made of a parylene–hydrogel double-layer system with high water retention over extended periods (six months). This type of hydrogel was able to detect skin hydration, temperature changes alongside other electrophysiological signals based on the temperature-dependent conductivity, and the sol–gel transition of the gelatin component of the hydrogels.

In summary, the self-healing hydrogels developed to date can usually only achieve self-repair in a static environment or under specific conditions because the dynamic non-covalent bonds and reversible covalent bonds are highly sensitive to external environments such as pH, temperature, and solvents. It is difficult to achieve self-repair under frictionally dynamic conditions under current hydrogel design.

## 4. Conclusions and Outlook

It has been clearly demonstrated that the key working principles of environmentally responsive hydrogels used as water-based lubricants lie in their ability to form hydration films in the presence of physical or chemical elements embedded within the hydrogel that can modulate the thickness of these films in response to environmental changes. The adsorption and stabilization of the polymer chains within the hydrogel on the surface of the friction pair are critical to the effectiveness of hydrogels as water-based lubricants.

Polymeric hydrogels can be engineered to respond to a wide range of external stimuli, including changes in electric fields, temperature, light, pH, shear stress, and more. Various physical structures or chemical groups can be incorporated into the hydrogel polymer chains to trigger the following mechanisms:

Electric Field Response: the directional migration of hydrophilic groups or charged molecules within the hydrogel under an electric field alters the thickness of the surface hydration layer or the arrangement of lubricating molecules.

Temperature Response: the use of temperature-responsive polymers enables the rearrangement of hydrophobic and hydrophilic groups, which affects the friction coefficient through changes in molecular chain adhesion on the friction surface.

Light Response: light can either be converted into heat to activate temperature-responsive polymers or induce molecular conformational changes. These changes can alter the hydrophilic/hydrophobic balance, charge distribution, or swelling state of the polymer network, thereby regulating the thickness and friction coefficient of the interfacial hydration layer.

pH Response: variations in pH can trigger the protonation or deprotonation of ionizable groups on the hydrogel chains, leading to changes in network charge, swelling behavior, and surface hydration properties. These changes influence interfacial friction and hydration layer characteristics.

Shear Stress Response: shear thinning behavior or the dissociation of non-covalent supramolecular chains within the hydrogel can lead to the release or directional alignment of interfacial lubricants, significantly reducing the static friction coefficient.

Although the research on responsive lubricating hydrogels has made great progress, it is still in a relatively preliminary stage in terms of design ideas, preparation methods, and applications in the harsh and variable engineering environment. There are few commercially available products in the markets. The main barriers or unresolved challenges relate to the scalability, durability, response times, and economic feasibility of the stimuli-responsive hydrogel and the composite lubricants for the heavy-duty engineering application. There are fundamental questions concerning mechanical properties, phase transition behavior, and multi-stimuli interactions. Determining the lubrication behavior and materials from native creatures and constructing composite materials with hydrogels embedding to friction-pair materials, which maximize the adsorption and stabilization of the hydrogel’s molecules on the surface of friction pair, could be a feasible route for the wide application of hydrogels as water-based lubricants. There are broad demands for water-based lubricants not only for ships, but also for soft robots, soft actuators, and intelligent robots. In-depth explorations and research are still needed in the design strategies of hydrogel-based lubrication and the precise regulation of their responses. Future research can be guided by the lubrication mechanisms of environmentally responsive hydrogels and introduce this influence into the polymerization framework. Then, the preparation of environmentally responsive lubricating hydrogels can be achieved using responsive supramolecular systems or dynamic covalent bond systems. The obtained hydrogels not only exhibit responsiveness to multiple factors but also have good mechanical properties when they are used as lubricants in underwater equipment.
